# Machine-Learned Association of Next-Generation Sequencing-Derived Variants in Thermosensitive Ion Channels Genes with Human Thermal Pain Sensitivity Phenotypes

**DOI:** 10.3390/ijms21124367

**Published:** 2020-06-19

**Authors:** Jörn Lötsch, Dario Kringel, Gerd Geisslinger, Bruno G. Oertel, Eduard Resch, Sebastian Malkusch

**Affiliations:** 1 Institute of Clinical Pharmacology, Goethe-University, Theodor-Stern-Kai 7, 60590 Frankfurt am Main, Germany; kringel@med.uni-frankfurt.de (D.K.); geisslinger@em.uni-frankfurt.de (G.G.); malkusch@med.uni-frankfurt.de (S.M.); 2 Fraunhofer Institute for Molecular Biology and Applied Ecology IME, Branch for Translational Medicine and Pharmacology TMP, Theodor-Stern-Kai 7, 60596 Frankfurt am Main, Germany; bruno.oertel@me.com (B.G.O.); Eduard.Resch@ime.fraunhofer.de (E.R.); 3 Fraunhofer Cluster of Excellence for Immune Mediated Diseases (CIMD), Theodor-Stern-Kai 7, 60590 Frankfurt am Main, Germany

**Keywords:** next-generation sequencing, human genomics, pain, experimental pain models, data science, machine learning

## Abstract

Genetic association studies have shown their usefulness in assessing the role of ion channels in human thermal pain perception. We used machine learning to construct a complex phenotype from pain thresholds to thermal stimuli and associate it with the genetic information derived from the next-generation sequencing (NGS) of 15 ion channel genes which are involved in thermal perception, including *ASIC1*, *ASIC2*, *ASIC3*, *ASIC4*, *TRPA1*, *TRPC1*, *TRPM2*, *TRPM3*, *TRPM4*, *TRPM5*, *TRPM8*, *TRPV1*, *TRPV2*, *TRPV3,* and *TRPV4*. Phenotypic information was complete in 82 subjects and NGS genotypes were available in 67 subjects. A network of artificial neurons, implemented as emergent self-organizing maps, discovered two clusters characterized by high or low pain thresholds for heat and cold pain. A total of 1071 variants were discovered in the 15 ion channel genes. After feature selection, 80 genetic variants were retained for an association analysis based on machine learning. The measured performance of machine learning-mediated phenotype assignment based on this genetic information resulted in an area under the receiver operating characteristic curve of 77.2%, justifying a phenotype classification based on the genetic information. A further item categorization finally resulted in 38 genetic variants that contributed most to the phenotype assignment. Most of them (10) belonged to the *TRPV3* gene, followed by *TRPM3* (6). Therefore, the analysis successfully identified the particular importance of TRPV3 and TRPM3 for an average pain phenotype defined by the sensitivity to moderate thermal stimuli.

## 1. Introduction

Thermal pain induced by heat or cold stimuli has been involved in various different clinical types of pain, such as inflammatory conditions [1,2]; peripheral nerve injury [3], including chemotherapy-induced neuropathic pain [4]; or painful bladder syndrome [5]. The importance of thermal pain is emphasized by the observation that human experimental pain models that use thermal nociceptive stimuli provide a comparatively good prediction of clinical analgesic drug effects [6,7]. For drugs targeting thermosensitive ion channels, heat or cold-triggered nociception is relevant in both the experimental and clinical setting [8].

The family of transient receptor potential (TRP) channels gated by thermal stimuli comprise different TRP ion channels (e.g., ankyrin (A), canonical (C), melastatin (M), vanilloid (V)) that have specific activation thresholds, ranging from noxious heat (TRPV2 ≥ 52 °C; TRPV1 ≥ 43 °C) via warmth (TRPM2 ≥ 37 °C; 30 °C ≤ TRPM3 ≤ 35 °C; 26 °C ≤ TRPV3, TRPV4 ≤ 34 °C) and cold (25 °C ≤ TRPC5 ≤ 37 °C; TRPM8 ≤ 25 °C) up to noxious cold (TRPA1 ≤ 17 °C) [9]. Further known cold sensors include different acid sensing ion channels (ASIC) (ASIC1 [10]; ASIC2; ASIC3 [11]; and, with a regulatory role for other ASICs, ASIC4 [12]). The ASIC ion channels are modulated by a cold temperature of ≤25 °C [11] and variants in their coding genes are primary candidate modulators of thermal nociception and therefore relevant for the variability of thermal pain and/or the effects of various novel analgesic drugs under clinical development [13] (Table 1).

The human perception of pain induced by thermal stimuli has been repeatedly addressed by genetic association studies, which, by identifying modulatory variants, decoded the relevant genes for the pain phenotype [14,15]. A recent assessment of human heat pain and its enhancement by topical capsaicin application showed by an association analysis of human genetic variants that TRPA1 plays a more important role in this pain-related phenotype than TRPV1 [15]. However, in addition to capsaicin, this study also included local ultraviolet (UV-B) irradiation as a second hypersensitizing procedure that not only seems to have influenced sensitivity to heat pain, which can be explained by the excitation of TRPV4 and TRPV1 [16,17], but also sensitivity to cold pain [18]. Therefore, in the present analysis, the role of several different thermosensitive ion channels in the modulation of thermal pain induced by either hot or cold stimuli was determined by the relative importance of variants in their coding genes, using next-generation sequencing (NGS) and the training of artificial intelligence to assign an individual to the correct thermal pain phenotype [15].

## 2. Results and Discussion

### 2.1. Participants and Descriptive Data

Of the total 100 participants in the study (age: range 19–42 years, mean ± standard deviation, SD: 25 ± 3.5 years, sex: 54 women), phenotyping was complete in a cohort of *n* = 82 subjects (age: range 19–33 years, mean ± standard deviation, SD: 24.7 ± 2.7 years, sex: 45 women). In the remaining subjects, the pain phenotypes were only partially recorded or not recorded at all, and since the key data were not imputed, these subjects had to be excluded from the analysis. Genetic information was available from *n* = 67 subjects due to failed quality checks in the remaining samples despite repeated NGS runs. The genetic information initially comprised 1070 variants with at least one deviation from the reference hg19 sequence, of which 78.7% were single nucleotide variants (SNV), 10.85% were deletions (Del), 8.6% were insertions (Ins), and 1.79% were mixed variants (MIX).

### 2.2. Phenotypes of Thermal Pain

**Figure 1:**
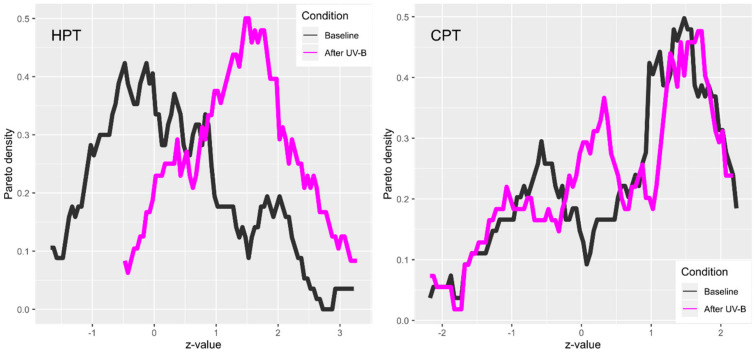
Distribution of the z-values of the quantitative sensory testing parameters heat pain threshold (HPT) and cold pain threshold (CPT), acquired at baseline (black) and following hypersensitization using the topical application of UV-B (violet). The density distribution is presented as probability density function (PDF), estimated by means of the Pareto Density Estimation (PDE [23]), as implemented in the R package “DataVisualizations” (https://cran.r-project.org/package=DataVisualizations [24]). Higher z-scores indicate a higher sensitivity to the respective stimuli. The figure has created using the R software package (v. 3.6.2 for Linux; http://CRAN.R-project.org/ [25]) and the libraries “ggplot2” (https://cran.r-project.org/package=ggplot2 [26]).

An examination of the pain thresholds determined on the control side or after UV-B irradiation showed that a transformation of the data was not necessary. The phenotypic analyses were therefore performed in the data obtained after the processing of the sensory thresholds according to the standard procedure recommended by the German Research Network for Neuropathic Pain [20,21,22]. A plot of the distribution of pain thresholds to thermal stimuli showed that for heat stimuli, UV-B caused a clear shift towards higher sensitivity, whereas for cold stimuli the effect was less pronounced (Figure 1). The shape of data distribution was changed, but only a moderate tendency towards a shift to the right was observed.

**Figure 2:**
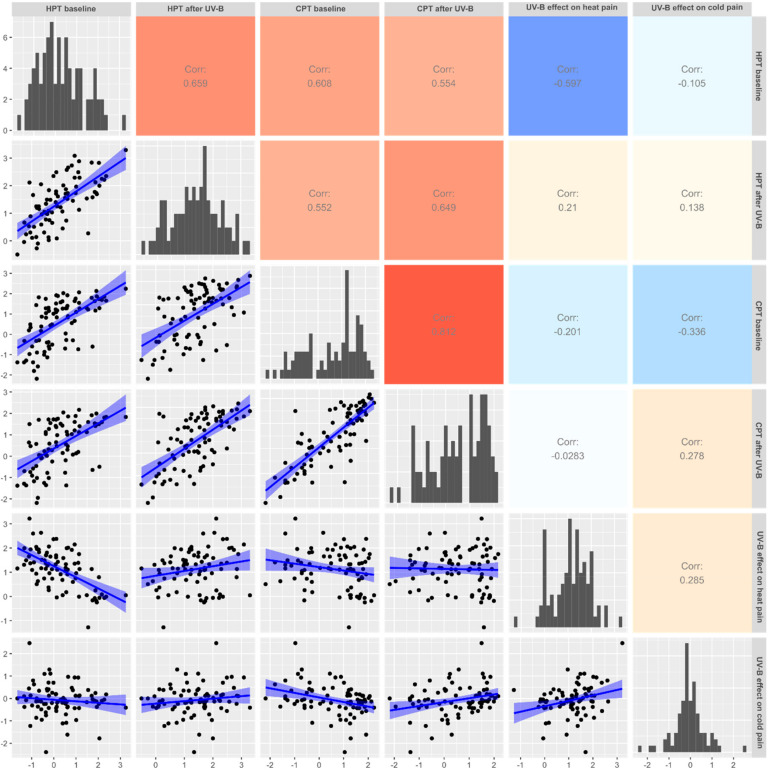
Correlation scatter plot matrix of the pain thresholds to heat and cold stimuli acquired under control conditions or after UV-B irradiation, and of the UV-B effects calculated as the differences between the corresponding thresholds with and without UV-B-induced hypersensitization. In the lower left part, single observations are shown as black dots. The lines indicate a linear fit (and confidence intervals) for visual guidance. Please note, however, that the statistics have been done using non-parametric correlations shown in the upper right part of the matrix as values of Pearson’s *r*. The correlation strength is also color coded from red, indicating a high positive correlation, via grey/white, indicating no correlation, to blue, indicating a strong negative correlation. The diagonal shows the data distribution as histograms. The figure has created using the R software package (v. 3.6.2 for Linux; http://CRAN.R-project.org/ [25]) and the libraries “ggplot2” (https://cran.r-project.org/package=ggplot2 [26]) and “GGally” (https://cran.r-project.org/package=GGally [27]).

The correlation matrix among the features related to thermal pain and the UV-B effects (Figure 2) indicated a strong correlation between the two pain thresholds to cold stimuli, *zCPT_baseline_* and *zCPT_UVB_* (Pearson’s *r* = 0.812, *p* < 2.2 × 10^−16^). The PCA of these two parameters resulted in two principal components with eigenvalues of 1.81 and 0.188, explaining 90.58 and 9.42 of the total variances, respectively. For the first component, *PC1_cold_*, *zCPT_baseline_* and *zCPT_UVB_* contributed equally. *PC1_cold_* was used in the cluster analyses instead of the two cold pain thresholds.

**Figure 3:**
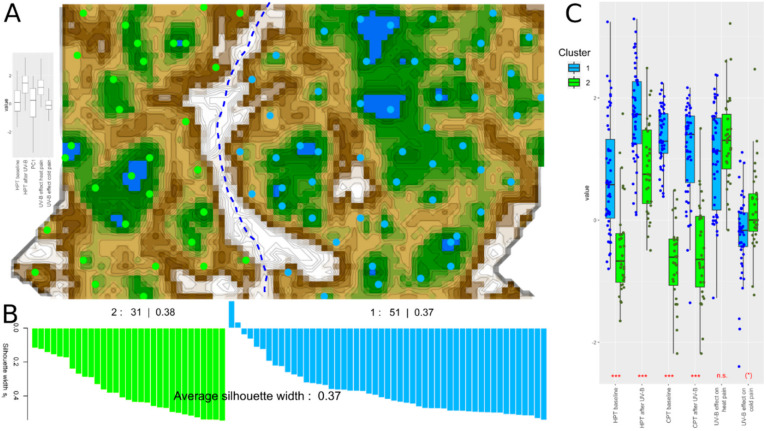
Neural network (emergent self-organizing maps (ESOM)/U-matrix)-based clustering [28] of the pain thresholds to thermal stimuli and the effects of UV-B irradiation, exploring the 82 × 5-sized matrix composed of the values of *zHPT_baseline_, zHPT_UVB_, UVBEff_Heat_*, and *UVBEff_cold_* acquired or calculated in 82 subjects, and in addition to *PC1_cold_* being the first component of a PCA of the highly correlated parameters *zCPT_baseline_* and *zCPT_UVB_*. (**A**) U-matrix visualization of distance-based data structures, using a projection of the data points onto a toroid grid of 4000 neurons where opposite edges are connected. The dots represent the so-called “best matching units” (BMU)—i.e., neurons on the grid that after ESOM learning carried the vector that was most similar to a subjects’ data vector. The U-matrix visualization was colored as a top view of a topographic map with brown (up to snow-covered) “heights” and green “valleys” with blue “lakes”. Watersheds indicate borderlines between different clusters. Two clusters emerged in this way, separated by the white “mountain ridge” in the center of the U-matrix. BMUs belonging to clusters #1 or #2 are colored in blue or green, respectively. Some heterogeneity within the cluster can be observed, however without providing a clear separation of a further cluster. The box plot panel to the left of the U-matrix displays the variables submitted to the ESOM-based data projection. (**B**) Silhouette plot [29] indicating how near each subject was to its own cluster relative to the neighboring clusters. Positive values indicate that the sample is away from the neighboring clusters, while negative values indicate that those samples might have been assigned to the wrong cluster because they are closer to the neighboring cluster than to their own cluster. (**C**) Pattern of pain thresholds among the two pain-related phenotypes resulting from the cluster analysis (***: *p* < 0.001 (uncorrected). (*): *p* < 0.05 (uncorrected)). The boxes have been constructed using the minimum, quartiles, median (solid line within the box), and maximum. The whiskers add 1.5 times the inter-quartile range (IQR) to the 75th percentile or subtract 1.5 times the IQR from the 25th percentile. The dots indicate single data points, jittered to enhance visibility. Alpha-corrected significant group differences according to paired *t*-tests are indicated at the bottom of the box pairs. The figure has been created using the R software package (v. 3.6.2 for Linux; http://CRAN.R-project.org/ [25]), and the R libraries “Umatrix” (https://cran.r-project.org/package=Umatrix [30]), “cluster” (https://cran.r-project.org/package=cluster [31]) and “ggplot2” (https://cran.r-project.org/package=ggplot2 [26]).

After a distance-preserving projection of the data from high-dimensional space onto a grid of 50 × 80 artificial neurons and the addition of the distances in the high-dimensional space as a color code, a clear two-cluster structure was found in the dataset (Figure 3A). Specifically, the U-matrix visualization resulted in two different clusters of *n_1_* = 51 and *n_2_* = 31 subjects, which were visually separated from each other on the topographic map analogy by a “snow-covered mountain ridge”. The cluster solution had silhouette indices of 0.37.

Repeated incremental clipping for error reduction (RIPPER) was able to assign the subjects perfectly with 100% accuracy to the correct cluster. These rules consisted initially of three rules that could be combined into a single rule reading “*IF zCPT_baseline_ ≤ 0.19 OR zHPT_baseline_* ≤ *−1.06 THEN the subject belongs to cluster #2 ELSE the subject belongs to cluster #1*”. Nevertheless, the clusters differed in further pain-related parameters (Figure 3C), as indicated by significant effects in the rm-ANOVA (main effect “test”: *df* = 5400, *F* =, 54.48, *p* = 4.3 × 10^−43^; effect “cluster”: *df* = 1,80, *F* = 112.922, *p* = 5.9 × 10^−6^ interaction *t*-tests (*zHPT_baseline_*: *t* = 5.9434, *df* = 72.274, *p* = 9.041 × 10^−8^, *zHPT_UVB_*: *t* = 5, *df* = 63.665, *p* = 4.77 × 10^−17^; interaction “test” by “cluster”: *df* = 5400, *F* = 45.212, *p* = 5.8 × 10^−37^. Post-hoc *t*-tests for the cluster differences resulted in *zCPT_baseline_*: *t* = 15.741, *df* = 49.529, *p* < 2.2 × 10^−16^, *zCPT_UVB_*: *t* = 9.1418, *df* = 58.381, *p* = 7.298 × 10^−13^, *UVBEff_Heat_*: *t* = −1.657, df = 69.417, *p* = 0.102, *UVBEff_cold_*: *t* = −2.6313, *df* = 59.316, *p* = 0.01082), of which the raw thresholds differences were all significant, while for the UV-B effects calculated as the differences between the baseline and post-irradiation thresholds, those on heat were not significant and those on cold were significant only at the uncorrected α level while exceeding the corrected α level of 0.00833. The sexes were equally represented in both clusters (χ^2^ = 0, df = 1, *p* = 1).

The present pain-related phenotypes were defined by the sensitivity to painful heat and cold stimuli at baseline. While UV-B had a pronounced effect on heat pain, it mainly provided a right-shift to higher sensitivity, which was probably captured in the clusters by the correlation with the baseline pain thresholds. However, other than previously observed with capsaicin-induced hypersensitization [15], the magnitude of the response to UV-B did not further divide the subjects into subgroups. Moreover, compared with heat pain, the UV-B-induced change in sensitivity was much less pronounced in cold pain and consisted mainly in a change in the shape and modality of the distribution rather than in a pronounced right shift. The change in the shape of the distribution explains why, in a slightly differently designed analysis that—in addition to thermal stimuli—included also several different mechanical nociceptive stimuli, a UV-B effect on cold pain was observed [18], triggering the present analytical design to include both heat and cold stimuli. This had the consequence of an average pain phenotype defined by the sensitivity to moderate thermal stimuli, in contrast to more extreme pain phenotypes that are often sought when using hypersensitization to heat or cold pain. Translated to the clinical setting, this may model patients who suffer from pain under average conditions without the need for an extreme hot or cold environment to trigger the pain. Which clinical setting is modelled is a decision to be made in experimental pain research.

Corresponding to the definition of the present pain phenotype by moderate cold and heat pain criteria, the genes carrying most variants identified as informative for phenotype association codes for thermal sensors with activation temperatures of 30 °C ≤ TRPM3 ≤ 35 °C and 26 °C ≤ TRPV3 [32,33]. This is compared with the other ion channels genotyped in this study nearest to the starting point of the thermal stimulation at 32 °C. The *TRPV3* gene variants were also among the most highly evaluated in terms of their importance for the subjects’ assignment to the pain phenotype. (Table 2). TRPV3 has also been found among the novel analgesic targets, in addition to other analgesics in development targeting TRPV1, with most hits for ASIC1, ASIC2, and TRPA1. However, this list lacked TRPM3, which appears not to have been considered in drug development to date, but is supported in the present analysis as a new target for analgesics.

### 2.3. Ion Channel Gene Variants Relevant to Phenotypes of Thermal Pain

After the elimination of variants with distributions of homozygous and heterozygous carriers of the respective alleles that did not meet the expectations from the Hardy–Weinberg equilibrium, *d* = 894 variants were in the dataset for genetic association analyses, of which 861 were in the candidate genes while the rest was in adjacent DNA positions. Most of them were very rare and, following the elimination of uninformative variants based on Shannon information content, *d* = 250 variants remained. Their allelic frequency was on average 20% (standard deviation 15%), with a range between 3% and 54%. Following the selection of variants based on the χ^2^ effect size for group differences between the pain phenotype clusters, *d* = 80 genetic variants were retained for the machine learning-based association analysis.

Thus, the data space for random forest-based classification was $D=\left\{\left(x_{i},y_{i}\right)|x_{i,d}\in \;X,\;y_{i}\in Y,\;i=1\ldots n\right\},$ with *d* = 80 genetic features acquired from *n* = 67 subjects belonging to *y* = 2 classes of pain phenotypes (pain cluster #1: *n* = 42, cluster #2: *n* = 25). Class assignment was achieved at a moderate performance, with a balanced accuracy of 58.5% (95% confidence interval, CI: 51.7–77.1%) and an area under the receiver operator characteristic curve (AUC-ROC) of 77.2% (95% CI: 66.3–86%). However, this certainly exceeded the accuracy obtained when training the random forests with permuted genetic information, where the genotype–phenotype relationship was deliberately destroyed and the classification was not better than by guessing—i.e., with a balanced accuracy of 50% (95% CI: 50–54.8%) and an AUC-ROC of 56.7% (95% CI: 45.7–75.3%). Furthermore, none of the *d* = 51 genetic variants in the negative control genes *CYP2J2*, *CYP2C9,* and *CYP2C19* had been selected among the most important variants in a separate analysis. It was concluded that NGS-based genotypes provided relevant information to assign a person to the correct pain-related phenotype.

**Figure 4:**
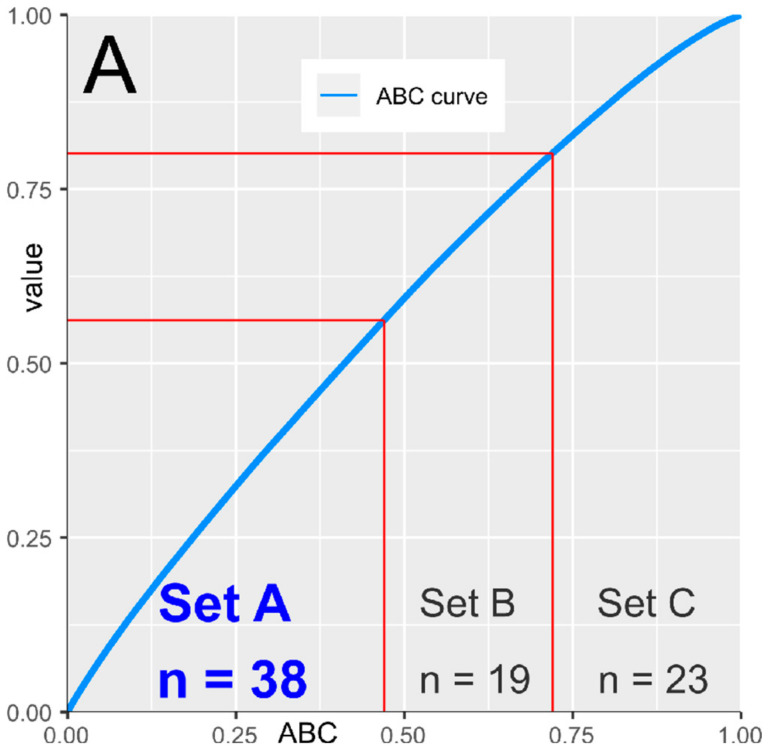
Identification of the most informative gene loci for pain phenotype class assignment using the computed ABC analysis. The mean decrease in classification accuracy associated with each genetic variant in the random forest analysis was submitted to ABC analysis, which is an item selection procedure aiming at the identification of the most profitable items from a larger list of items. The ABC plot (blue line) shows the cumulative distribution function of the mean decreases in accuracy (for further details about the computed ABC analysis, see [36]). The figure has been created using the R software package (v. 3.6.2 for Linux; http://CRAN.R-project.org/ [25]) and the R libraries “ABCanalysis” (http://cran.r-project.org/package=ABCanalysis [36]) and “ggplot2” (https://cran.r-project.org/package=ggplot2 [26]).

The computed ABC analysis identified *d* = 38 genetic variants that contributed most to the class assignment—i.e., belonged to the ABC set “A” (Figure 4).

**Figure 5:**
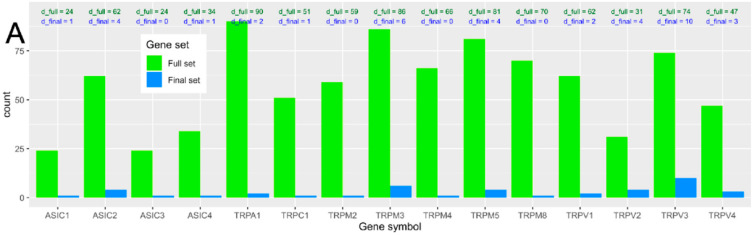
Display of the number of variants in the genes selected as candidates for the present association analysis. (**A**) Bar plot of the number of variants per gene included in the analysis. The full set (“d_full”, green bars) refers to the original set of genetic variants observed by means of the next-generation sequencing of the selected genes. The final set (“d_final”, blue bars) comprises the most informative variants that remained after several steps of feature selection and machine-learned association of the NGS-based genetic information with the machine learning-derived thermal pain-related phenotype. The number of variants per gene is also displayed numerically above the respective bar. The figure has been created using the R software package (v. 3.6.2 for Linux; http://CRAN.R-project.org/ [25]) and the R package “ggplot2” (https://cran.r-project.org/package=ggplot2 [26]).

Most of them (*d* = 10) belonged to the *TRPV3* gene, followed by *TRPM3* (*d* = 6), *TRPV2*, *TRPM5, ASIC2* (*d* = 4), *TRPV4* (*d* = 3), *TRPV1*, *TRPA1* (*d* = 2) *TRPC1*, *ASIC1,* and *ASIC4*, in decreasing order of the number of relevant variants carried (Figure 5). None of the variants belonging to the final set of the most relevant modulators of the phenotype were in *TRPM8*, *TRPM4*, *TRPM2,* or *ASIC3*. The number of variants in the final set comprised on average 4.4% of the number of variants in the original set, with a broad range of 0–13.5%. The number of variants per gene in the ABC set “A” was not statistically significantly correlated with the initial number of variants found by NGS in the respective genes (*r* = 0.38, *p* = 0.16; Figure 5).

The biological consequences of the genetic variations found in the main analysis to be informative for pain-phenotype association were queried from several publicly available databases including the NCBI gene index database (http://www.ncbi.nlm.nih.gov/gene), GeneCards (https://www.genecards.org), the Short Genetic Variations database (dbSNP) (https://www.ncbi.nlm.nih.gov/snp), Ensembl (http://www.ensembl.org), UniProt (https://www.uniprot.org), and ClinVar (https://www.ncbi.nlm.nih.gov/clinvar; all accessed in January 2020). Of the 38 most informative variants, 20 were found in intronic areas, up- or downstream or in untranslated regions; 7 variants were located in prime sites or regulatory parts; and 11 were coding mutations, including 5 synonymous, 1 deletion and 1 insertion (Table 2).

In order to obtain earlier evidence of a functional role of the selected genetic variants—i.e., those in ABC set “A” in the main analysis—a PubMed database search (https://www.ncbi.nlm.nih.gov/pubmed/) was conducted in January 2020 using the string “(asic1 OR asic2 OR asic4 OR trpa1 OR trpc1 OR trpm3 OR trpm5 OR trpv1 OR trpv2 OR trpv3 OR trpv4) AND (pain* OR analgesi*) AND (polymorphis* OR varian* OR mutation*) NOT (mice OR mouse OR rodent OR drosophila) NOT review”. This yielded 96 hits. After excluding 29 publications that reported results in laboratory animals, 65 studies remained. According to this, the variants in the relevant genes had been involved in eight different clinical situations related to pain (Table 3). A considerable number of the presently identified variants are supported by previous evidence that they are informative for the assignment of subjects to a pain-related phenotype in eight different clinical situations related to pain (Table 3). In brief, one of the most frequently evaluated variants was the loss of function mutation rs8065080 (I585V), which is associated with an enhanced pain threshold and decreased sensitivity to noxious and innocuous stimuli [37].

Despite that, rs8065080 was associated with neuropathic pain [38,39], painful osteoarthritis [40], and heat pain sensitivity in humans [41]. Furthermore, another highly ranked *TRPV1* variant, rs4790522 in *TRPV1,* was associated with thermal pain sensitivity [41] and asthma [42,43]. A genome-wide association study (GWAS) analysis in rheumatoid arthritis patients revealed that the *TRPV1* variant rs1039519 was the most strongly associated single nucleotide polymorphism (SNP) with the highest *p*-value (7.11 × 10^−5^) on chromosome 17 [44]. The *TRPA1* polymorphism rs3735943 was reportedly associated with pain crisis in sickle cell disease [45] and with sensitivity to heat stimuli and topically applied capsaicin [15], the latter in the same cohort as that presently analyzed. Other hits were variants rs8121 and rs395357 in the *TRPV2* and *TRPV3* genes, respectively, associated with the genetic risk of fibromyalgia, a disease characterized by widespread musculoskeletal pain, in a Korean cohort [46].

Apart from the pain context, the variants included in the current selection of the *d* = 38 most informative gene loci are additionally supported by previous evidence of a functional role in other clinical situations. For example, an association of the *TRPM5* polymorphism rs4929982 with primary open angle glaucoma has been reported [47], and carriers of the *ASIC2* polymorphism rs28936 had a higher risk of developing panic disorder [48] or multiple sclerosis [49,50], while the *ASIC4* mutation rs11695248 was associated with paroxysmal dystonia [51].

Further information on the possible biological significance of the most relevant genetic variants assigned to the ABC set “A” was obtained by assessing the variants’ so-called Eigen score [35]. The Eigen score is defined as a functional variant-importance score that includes the information of several different functional genetic annotations. It uses a spectral unsupervised approach that assigns weights to each functional annotation based on its predictive accuracy. The functional score is then calculated from the optimized weighted linear combination of the annotations. Since the relevance of the annotations varies between coding and non-coding variants, their Eigen scores are calculated separately. Due to its unsupervised nature, it is not based on a priority assumption.

For a comparative interpretation of the biological significance of the variants in the ABC set “A”, a positive control set was created from 128 variants in five different genes causally involved in familial syndromes with pain insensitivity—i.e., *SPTCL*, *WNK1*, *TTR*, *SCN9A,* and *SCN11A*. All the variants were declared “pathogenic” or “probably pathogenic” by the online tool Variation Viewer (https://www.ncbi.nlm.nih.gov/variation/view/; assessed in January 2020), and the Eigen scores for all possible mutations comprised a total of 384 variants. In addition, a set of 1232 variants randomly drawn from the Eigen database (v. 1.1, http://www.columbia.edu/~ii2135/eigen.html) throughout the hg19 served as a negative control set of variants. The Eigen score distributions of the three sets of variants were compared against each other using Wilcoxon–Mann–Whitney U-tests [52,53] implemented in the R-”stats” base library.

**Figure 6:**
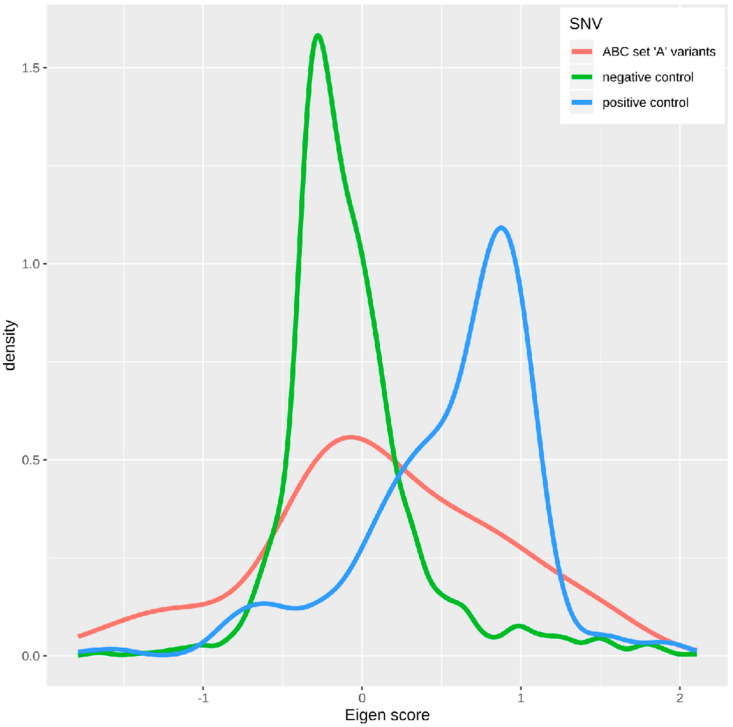
Eigen score distribution of functional genetic variants. Density plot of the distribution of Eigen scores for three different datasets of single nucleotide variations (SNV). Red line: variants assigned to the ABC set “A” in the feature selection (*d* = 36; two variants not in the Eigen database). Blue line: variants causally involved in hereditary syndromes with insensitivity to pain. Green line: random sample drawn from a generic dataset comprising Eigen scores of variants across the human genome queried Eigen score database (http://www.columbia.edu/~ii2135/eigen.html). The figure has been created using the R software package (http://CRAN.R-project.org/ (R Development Core Team, 2008)) and the library “ggplot2” (https://cran.r-project.org/package=ggplot2 (Wickham, 2009)). The density distribution was smoothed using a gaussian kernel.

The distribution of the Eigen score values obtained for the variants in the ABC set “A” (mean ± standard deviation, SD: 0.123 ± 0.751, *d* = 36, as two variants were missing from the Eigen database; red line in Figure 6) was shifted to the right—i.e., to higher Eigen scores—from the negative control dataset obtained from a completely random selection of variants throughout hg19 (Eigen score = −0.068 ± 0.433, *d* = 1232; green line in Figure 6). The difference was statistically significant (Wilcoxon *W* = 17190, *p* = 0.021). However, the right shift was modest compared to the distribution of the Eigen scores of the variants causally associated with hereditary syndromes with pain insensitivity (Eigen score = 0.557 ± 0.552, *d* = 384; blue curve in Figure 6). Their Eigen scores differed statistically significantly from those of the random sample of variants (*W* = 80302, *p* = 2.861 × 10^−85^) but also from those of the variants in the ABC set “A” (*W* = 9571, *p* = 0.135 × 10^−4^). The variants in the ABC set “B” (Eigen score = −0.031 ± 0.501, *d* = 19; data not shown) did not significantly alter from the negative control data (*W* = 9532, *p* = 0.163), but were significantly left-shifted compared to the positive control data (*W* = 5898, *p* = 5.66 × 10^−6^). The variants in the ABC set “C” (Eigen score = 0.091 ± 0.793, *d* = 23; data not shown) did not significantly alter from the negative control data (*W* = 12727, *p* = 0.403), but were significantly left-shifted compared to the positive control data (*W* = 6317, *p* = 5.24 × 10^−4^).

### 2.4. Strengths and Limitations

The data analysis followed the idea that an artificial intelligence trained with genetic information that outperforms the pain-phenotype assignment of unknown subjects by random indicates that the genotypes comprise relevant information on the phenotype. This was observed in the present data analysis. When the genotype–phenotype association in the training dataset was destroyed by the permutation of the individual genotypes, the phenotype assignment was deteriorated to the level of randomness. Successful AI training with the true genetic information and unsuccessful training with the false genetic information was observed repeatedly on randomly drawn subsamples of subjects, indicating that the observation of a genotype–phenotype association was robust. The genetic information of genes that, according to current mechanistic knowledge, had no association with the pain phenotypes, was correctly discarded during a data analysis with additional NGS data, while the finally selected reduced set of variants was located in genes with a biologically highly plausible association with the pain phenotypes.

Thus, the data analysis used intentionally supervised machine learning for knowledge discovery rather than for the creation of a working AI-based diagnostic tool. Its results successfully supported the association of the genetic information with the recognized pain phenotypes. Here, however, machine learning was stopped at that point because the genetic background of pain comprises many more genes—currently about 540 [54,55]—so it is unlikely that the present genes could provide a perfect assignment of subjects to the pain phenotype. The modest accuracy and receiver operator characteristic curve (ROC) area achieved for the assignment of phenotypes is probably not due to the poor selection or implementation of the AI, but rather reflects the truth about the small phenotypic effects of the ion channel genotypes. This corresponds to earlier observations, where the genetic variants found in average healthy individuals had much smaller effects on the pain thresholds than, e.g., hypersensitization by topical capsaicin application [56]. A comparison with variants found in rare cases of familial insensitivity to pain supports this hypothesis. These variants had much higher Eigen scores quantifying the biological effects of a DNA sequence change than the set of genetic variants resulting from the present analysis (Figure 6).

Finally, it has to be acknowledged that the genotype versus phenotype association did not include an independent validation cohort. Partly accounting for this lack are (i) the a cross-validation approach and (ii) the fact that the gene set had been selected based on evidence for a role in temperature sensation and pain, indicating that this analysis was not an attempt to find a novel genetic association but rather a ranking of genes and genetic variants with respect to the phenotypic importance. Moreover, (iii) several of the variants identified as important in the present phenotypic setting are supported for a phenotypic role by independent previous publications cited above.

## 3. Methods

### 3.1. Subjects and Study Design

In the present study, *n* = 100 healthy volunteers (46 men) of Caucasian ethnicity by self-assignment were enrolled at the age of 19–42 years (mean value ± standard deviation 25 ± 3.5 years), as described previously [18]. The study followed the Declaration of Helsinki and was approved by the Ethics Committee of the Medical Faculty of the Goethe-University Frankfurt am Main, Germany (protocol number 28/11, approved on 3 March 2011). All the subjects gave their informed written consent to the study procedures, including genotyping. Phenotypic assessments and a genetic association approach in the dataset generated in this study were published previously [15,18] in a non-redundant manner to the present analyses.

The inclusion criteria were age between 18 and 50 years, actual health according to anamnesis, and the physical examination of vital parameters. The exclusion criteria were medications taken during the previous week, except for oral contraceptives, vitamins, or hormone substitutes. The further exclusion criteria were a current clinical condition with pain and current illnesses according to the survey or the medical examination. Alcohol consumption was prohibited for 24 h prior to the trials. Before the actual experiments, all the subjects underwent training to familiarize them with the pain model. During this session, pain tests were conducted in areas other than those used in the experiments.

### 3.2. Assessment of Thermal Pain Thresholds

The recording of sensory thresholds for different stimuli has already been described in detail [18]. It was performed in strict compliance with a standard procedure developed by the German Research Network for Neuropathic Pain [20,21]. Room temperature was maintained at 20–25 °C during the tests. For the present genetic assessments, pain thresholds to heat and cold stimuli were selected based on the previous finding that both thresholds were affected by UV-B irradiation [18]. The thermal pain thresholds were determined using a 3 × 3 cm thermode (TSA 2001-II, Ramat Yishai, Israel) on a 9 cm² skin area on the inside of the forearm without superficial veins or moles. The heat pain thresholds (HPT) were measured by increasing the temperature of the thermode by 1 °C/s, starting at 32 °C, until the test subject indicated pain, which triggered the reversal of the temperature ramp back to the baseline. According to the published guidance for the quantitative sensory testing (QST) test battery [20,21,57], the HPT was defined as the mean value of three repeated measurements. The cold pain thresholds (CPT) were recorded analogously, with the exception that the temperature of the thermode was lowered by 1 °C/s from 32 °C to a cut-off temperature of 0 °C.

The UV-B pain model uses ultraviolet light to induce a small area of inflammation, which allows an assessment of mechanical and thermal thresholds [58,59,60]. To determine the minimum erythema dose, six areas of 1 cm² were first irradiated with a cumulative UV-B dose between 200 mW/cm² and 600 mW/cm² (UV-B lamp UV 109 from Waldmann Medizintechnik, Villingen-Schwenningen). The lamp was placed 2.5 cm from the skin. The UV-B dose was increased by extending the irradiation time. The minimum erythema dose was determined to be the smallest dose that led to a visual reddening of the irradiated skin area after 24 h. Subsequently, a previously unirradiated skin area on the inner forearm was selected for the actual experiments according to the same criteria as for the determination of the minimum erythema dose. This area was irradiated with twice the determined amount, and the actual experiments were performed 24 h after UV-B irradiation.

According to the standard protocol of the German Research Network for Neuropathic Pain [20,21], the quantitative sensory data were z-transformed into a reference group of 180 healthy subjects, from which a dataset of 1080 values was determined [22]. According to the standard protocol, the z-transformation was performed separately for the test field, sex, and age of the subjects—i.e., the procedure implies a correction for these factors. In addition, the thresholds for warmth stimuli—i.e., the temperatures at which the subjects indicated pain—were multiplied by a value of −1 to obtain a uniform direction of all values, with larger values indicating high pain sensitivity. Furthermore, the effect of UV-B on the thresholds was quantified as the difference between the measurement after UV-B application and the measurement without the presence of UV-B—i.e., $UVBEff_{Heat}=zHPT_{UVB}-zHPT_{baseline}$ for heat pain and $UVBEff_{Cold}=zCPT_{UVB}-zCPT_{baseline}$ for cold pain.

### 3.3. Genotyping Using Next-Generation Sequencing

The NGS of the coding regions of 15 ion channel genes including 12 ion channels with specific activation thresholds are placed within a moderate temperature interval (*T* = [25 °C; 37 °C], *ASIC1*, *ASIC2*, *ASIC3*, *ASIC4*, *TRPC1*, *TRPM2, TRPM3*, *TRPM4, TRPM5*, *TRPM8, TRPV3* and *TRPV4*) and 3 ion channels with activation thresholds located in noxious regions (17 °C ≤ T or T ≥ 43 °C, TRPV1, TRPV2, TRPA1); this assay was performed using a custom AmpliSeq™ library and a validated assay on an Ion Torrent^TM^ personal genome machine (Thermo Fisher Scientific, Waltham, MA, USA), as described in detail previously [61]. In brief, genomic DNA was extracted from 200 µL venous blood on a BioRobot EZ1 workstation (Qiagen, Hilden, Germany) applying the blood and body fluid spin protocol provided in the EZ1 DNA Blood 200 µL Kit (Qiagen, Hilden, Germany). A multiplex amplification primer set for the exonic sequences of the ion channel genes was designed online using a web tool (Ion Ampliseq^TM^ Designer; Thermo Fisher Scientific, Waltham, MA, USA) provided by the manufacturer of the NGS device at http://www.ampliseq.com.

The present amplification design obtained a coverage of 97% of the target sequence. Following sequencing, signal processing was performed using the Torrent Suite software (v. 5.2.2; Thermo Fisher Scientific, Waltham, MA, USA), base calling and the generation of unmapped and mapped BAM-files (hg19 reference genomic sequence) were performed. Variant calling across the hg19 reference genomic sequence was performed with the Torrent Variant Caller Plugin (minimum quality = 10, minimum coverage = 20, and minimum coverage on either strand = 3), and variant annotation was performed using the Ion Reporter Software (v. 5.2.2; Thermo Fisher Scientific, Waltham, MA, USA). Variant call format (VCF) files containing the nucleotide reads were processed toward the individual genotypes using the GenomeBrowse^®^ software (v. 2.0.4, Golden Helix, Bozeman, MT, USA) and SNP and Variation Suite software (v. 8.7.1; Golden Helix, Bozeman, MT, USA).

### 3.4. Data Analysis

The data was analyzed using the R software package (v. 3.6.2 for Linux; http://CRAN.R-project.org/ [25]) on an Intel Core i9^®^ computer running Ubuntu Linux 18.04.3 LTS 64-bit (Canonical, London, UK)).

#### 3.4.1. Quantitative Variables

The pain data comprised the z-transformed pain thresholds to heat or cold stimuli recorded under control conditions after UV-B irradiation, and the UV-B effects were calculated as the difference between the z-transformed thresholds. This provided an n × 6 input data space $D=\left\{\left(x_{i}\right)|x_{i}\in \;X,\;i=1,\ldots ,n\right\}$, which included vectors *x_i_* = *<x_i_*,_1_,…,*x_i_*,*_d_>* with *d* = 6 different parameters representing the six different pain thresholds or UV-B effect-related variables (*zHPT_baseline_*, *zHPT_UVB_*, *zCPT_baseline_*, *zCPT_UVB_*, *UVBEff_Heat_*, and *UVBEff_cold_*) acquired from n subjects. The genetic data contained variables for all chromosomal locations where in at least one subject an allele had been found that that differed from the allele found at this location in the hg19 reference sequence. The variables were coded as [0,1,2], which indicates the number of alleles different from the hg19 reference allele at the respective chromosomal location.

#### 3.4.2. Data Analysis Strategy

**Figure 7:**
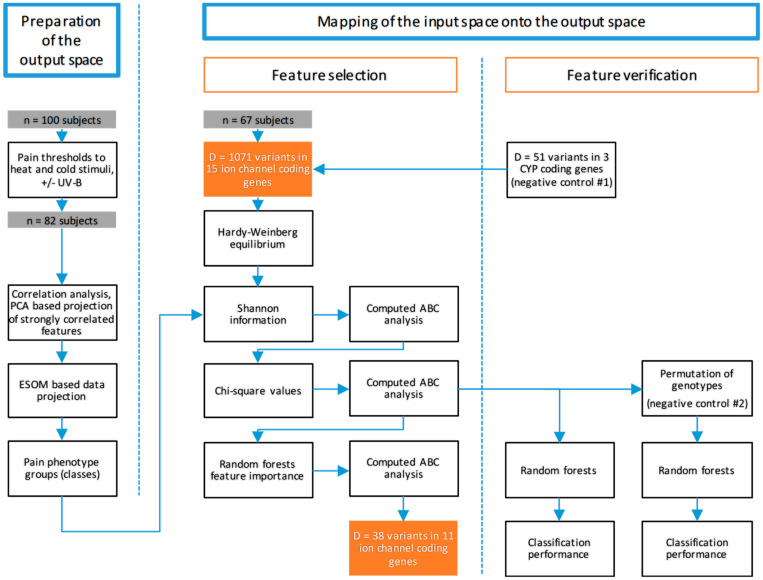
Flow chart of the data analysis. The figure gives an overview of the applied data science approach in two main steps (shown in blue: preparation of the output space, mapping of the input space with feature selection and verification of the genetic results). After the identification of a pain-related phenotype group structure (“output space”) based on thresholds for thermal pain and UV-B effects, genetic variants in the pre-selected candidate genes coding for different thermosensitive ion channels were selected to provide the most important information to train a machine-learned algorithm implemented as a random forest with the aim to assign a subject to the correct pain phenotype group. To verify the results, the correct group assignment was tested using randomly resampled data from the original dataset, on permuted data and including negative control genes present as cytochrome P450 (CYP) gene sequences.

The analysis aimed at mapping genotypic information onto phenotypic information. However, since the subjects participating in this study were healthy volunteers, the pain-related phenotypes had to be created first from the data obtained regarding pain threshold, thermal stimuli, and the effect of UV-B irradiation. The data analysis therefore used unsupervised methods to find structures in the pain-related data that support subgroups or clusters of subjects sharing similar sensitivity to thermal noxious stimuli. Subsequently, supervised machine learning was used to map the ion channel-related genetic information to these phenotypic results. An overview of the analyses is given in Figure 7.

#### 3.4.3. Establishment of Phenotypes of Thermal Pain

The phenotypes of thermal pain were evaluated in the data structures resulting from the z-transformed pain thresholds and the UV-B effects. This provided an 82 × 6 input data space including the *d* = 6 different parameters representing the six pain thresholds or UV-B effect-related variables (*zHPT_baseline_, zHPT_UVB_, zCPT_baseline_, zCPT_UVB_, UVBEff_Heat_,* and *UVBEff_cold_*) acquired from *n* = 82 subjects in who these data had been complete. In this input data space, the group structures were searched after checking the correlation structure of the data by of calculating Pearson’s *r* [62]. Strongly correlated features defined at a value of *r* > 0.8 were decorrelated using a principal component analysis (PCA) [63,64]). The relevant PCs, chosen as those with eigenvalues > 1 [65,66], were included in a clustering analysis for which a neural network in the form of emergent self-organizing maps (ESOM) [28] was used. This was preferred to classical clustering methods, based on previous demonstrations [28] that ESOM outperforms classical clustering methods in the detection of true cluster structures, as it does not impose a predefined shape on the clusters, which may with standard methods cause the occasional detection of spurious clusters in structureless data.

ESOM provide a distance-preserving method for projecting data from a high-dimensional space onto a low-dimensional grid, allowing the detection of structures in the data such as clusters or subgroups [67]. The maps were assembled from 50 × 80 = 4000 artificial neurons in the recommended standard size, which was implemented in our R-library “Umatrix” (https://cran.r-project.org/package=Umatrix [30]). Of note, this use of many artificial neurons differs from classical implementations of self-organizing aps where the neurons represent clusters and typically a small number of neurons is used. It can be shown that this type of self-organizing map (SOM) usage is identical to a k-means type of clustering algorithm [68]. In the present implementation, by contrast, SOMs are created where the map space is regarded as a tool for the characterization of the otherwise inaccessible high-dimensional data space. A characteristic of this SOM usage is the large number of neurons, and emergence in this regard is a precisely defined phenomenon of multi-agent systems [69].

A Gaussian-shaped neighborhood function and 20 epochs were used to train the ESOM. As a matter of fact, all the projections of high-dimensional space $R^{D}$ to lower dimensions $R^{d}$ with *d* << *D* must make mistakes, because the high-dimensional space simply does not fit into the low-dimensional space. The (generalized) U-matrix can be used to represent these errors [70] by showing the distances between the data points as hills on the initial plane. On the trained ESOM, therefore, the distance structure in the high-dimensional feature space was visualized with a U-matrix [28,71]. Colored with a topographic map analogy, large “heights” represent large distances in the feature space, low “valleys” represent similar data subgroups, and “mountain ranges” with “snow-covered” heights visually separate the clusters in the data [72]. As in hierarchical clustering, the silhouette index was calculated to assess the clustering quality. The better clustering solution between the two approaches was retained for the subsequent genotype association analyses. The quality of the clustering solutions obtained was evaluated using the silhouette index [29], which was calculated using the R-libraries “cluster” (https://cran.r-project.org/package=cluster [31]). Finally, the clusters were interpreted based on the original variables *zHPT_baseline_, zHPT_UVB_, zCPT_baseline_, zCPT_UVB_, UVBEff_Heat_,* and *UVBEff_cold_*. This was addressed by creating a set of rules implemented as repeated incremental clipping for error reduction (RIPPER [73]), as this type of hierarchical rule does not impose a restriction of binary splits. These calculations were performed with the R library “RWeka” (https://cran.r-project.org/package=RWeka [74]). The pain thresholds and UV-B effects were compared between clusters using an analysis of variance for repeated measures (rm-ANOVA), with “test” as the “within-subject factor” with five degrees of freedom, “cluster” as the “between-subjects factor”, and two-sample *t*-tests [75], with an α level set at 0.05 and corrected for multiple testing according to Bonferroni [76]. In addition, the unequal sex distribution among the pain phenotypes was assessed by means of a χ^2^ test [64].

#### 3.4.4. Mapping of Ion Channel-Related Genetic Information onto Thermal Pain Phenotypes

After establishing phenotypes, supervised machine learning was used to map the genetic information to the identified subgroup or cluster structures. The preprocessing of the genetic data included the elimination of variants where the distribution of homozygous and heterozygous carriers deviated from the expectation according to the Hardy–Weinberg equilibrium. This was assessed using Fisher’s exact tests with the R-package “HardyWeinberg” (https://cran.r-project.org/package=HardyWeinberg [77]).

Since NGS produced many candidate genetic variables and possibly irrelevant features in the input data space, dimension reduction was performed as the usual approach to narrow the focus on the most relevant variables for machine learning [78,79]. Thus, to avoid the inclusion of non-informative variants such as those carried by only very few subjects into the classifier, informative gene loci were detected based on the Shannon information [80] computed as $Info=-p_{0,i}\cdot \ln\left(p_{0,i}\right)-p_{1,i}\cdot \ln\left(p_{1,i}\right)\;$, where *p*_0,*i*_ and *p*_1,*i*_ are the observed probabilities of the non-observation (0) or observation (1), respectively, of a variant allele in the *i^th^* gene locus.

The exact limit of Shannon information up to which a gene locus can be considered sufficiently informative was calculated by means of a computed ABC analysis [36]. This is a categorization technique for selecting the most important subset from a larger set of positive items. It was chosen because it met the basic requirements of feature selection using filtering techniques [78]. That is, it easily scales to very high-dimensional datasets and is computationally simple, fast, and independent of the classification algorithm. The ABC analysis aims to divide a dataset into three unrelated subsets named “A”, “B”, and “C” [81]. The algorithm calculates the limits between these sets on the basis of the mathematical properties of the distribution of the analyzed items. Subset “A” contains the most profitable features [82,83]. The items of the set “A” were retained in further analyses. These calculations were performed using our R package “ABCanalysis” (http://cran.r-project.org/package=ABCanalysis [36]). Furthermore, as already implemented previously [84], further variants, which probably do not provide a suitable basis for the assignment of phenotype classes, were excluded due to the small effect sizes of the allele distribution between the phenotype classes. This was quantified using the classical χ^2^-statistics [64]. The χ^2^ values obtained for each gene locus were subjected to a computational ABC analysis as described above.

Following these feature-selection steps, the reduced data space $D=\left\{\left(x_{i},y_{i}\right)|x_{i,d}\in \;X,\;y_{i}\in Y,\;i=1\ldots n\right\}$ including the input space *X* comprising vectors *x_i_* = <x_i,1_,…x_i,d_> with *d* > 0 different parameters including the genetic information obtained from n = 82 cases of the output classes *y_i_*, comprising the pain-phenotype clusters identified in the first part of the data analysis, were submitted to supervised machine learning with the task of finding an assignment of the genetic information to the pain phenotypes. In random forests sets of different, uncorrelated and often very simple decision trees are created [85], with conditions on features such as vertices and classes such as leaves. Feature distributions are random, and the classifier refers to the majority vote for class membership provided by many decision trees. In this analysis, 1500 decision trees have been created with the number of features at 0.2·*sqrt(d)*. The number of trees and complexity were based on the smallest out-of-bag error rate obtained among various tested hyperparameter settings. Random forests were chosen as the classifier for the present analysis because previously they functioned similarly well on comparable genetic data as several different alternative machine learning algorithms, including adaptive boosting, k-nearest neighbors, naïve Bayes, support vector machines, and multivariate logistic regression [15,86].

Random forests learning was performed on 2/3 of the data randomly chosen by Monte–Carlo [87] resampling from the original data—i.e., the dataset was spilt into disjoint training and test data subsets using the R-library “sampling” (https://cran.r-project.org/package=sampling [88]). The trained classifier was then used to assign the subjects in the test dataset to their respective thermal pain-related phenotype. Using as many iterations as genetic variants, the mean decrease in classification accuracy when the respective variant was excluded from random forest learning was obtained for each genetic locus. The magnitude of this decrease indicated the importance of each genetic variant and was retained as the main result of this analysis. The most relevant genetic variants were identified by subjecting these values to a computed ABC analysis. The χ^2^ and random forests analyses were performed ten times on different data randomly resampled datasets to obtain robust results. In addition, to control for overfitting by the machine-learned algorithm, training was repeated with randomly permuted genetic data at each run. The classifier trained on this data should not be better than a guess, otherwise over-fitting was likely. Furthermore, two additional genes were included as negative controls in a separate analysis. Specifically, NGS-based genetic information about *CYP2J2*, *CYP2C9,* and *CYP2C19* was available for each subject from a different research context [89]. As no direct involvement in the modulation of nociception is known for these genes, the expectation for this analysis was that the chain of feature selection procedures would not select these variants to be members of the final set of informative variants for the thermal-pain-related phenotype.

The trained classifier was then used to assign the subjects in the test data subset to their respective thermal pain-related phenotype. The mean decrease in classification accuracy was maintained for each genetic locus when excluding the respective variant from random forest learning. The extent of this decrease indicated the importance of the respective genetic variant and was retained as the main result of this analysis. The most important genetic variants were identified by submitting these values to a computed ABC analysis. The χ^2^ and random forests analyses were performed 10 times on different datasets randomly resampled from the original data to obtain robust results. The classification performance was evaluated using the balanced accuracy and in addition the area under the receiver operator characteristic curve (AUC-ROC), calculated using the R libraries “pROC” (https://cran.r-project.org/package=pROC [90]) and “caret” (https://cran.r-project.org/package=caret [91]). These values were retained in each run and then averaged across the repetitions. In addition, to control for overfitting of the machine-learned algorithm, during each run the training was additionally performed with randomly permuted genetic data. The classifier trained on these data should not be better than guessing—i.e., provide balanced accuracy and AUC-ROC equal or close to 0.5—else overfitting of the random forests was likely.

## 4. Conclusions

The data presented here support a phenotype structure defined by the pain sensitivity to heat and cold at the baseline. Two phenotypes were found in which the assigned subjects had either a higher or lower sensitivity to heat and cold pain. This subgroup structure has a genetic basis, which lies in variants in thermosensitive ion channels involved in heat or cold sensation, among which those that perceive non-extreme temperatures appear to be particularly important. The clinical translation of the findings may be pain in the average environment rather than pain in situations of more extreme environmental exposure. This may have implications for drug development strategies that shift from targets involved in pain triggered by exceptional stimuli to targets triggered by multiple and moderate stimuli. In the present analysis, this has led to the highlighting of TRPM3 as a target for analgesics.

## Figures and Tables

**Table 1:** Novel analgesic drugs developed with the purpose of targeting proteins/genes associated with human hereditary insensitivity to pain—i.e., to mimic the pain insensitivity phenotype observed in carriers of loss of function mutations in these genes—that are currently in a clinical phase of development. The information was queried on January 2020 from the Therapeutic Target Database at http://db.idrblab.net [19].

Gene Symbol	Drug	Action	Company
*ASIC1*	PPC-5650	Modulator	Antalium
*ASIC2*	THA-904	Antagonist	Theralpha
*TRPA1*	HX-100	Inhibitor	Hydra Biosciences
*TRPV1*	CNTX-4975	Agonist	Centrexion Therapeutics
	DWP-05195	Antagonist	Daewoong Pharmaceutical
	GRC-15300	Antagonist	Glenmark Pharmaceuticals
	RESINIFERATOXIN	Activator	Sorrento Therapeutics
	ABT-102	Blocker	Abbott
	CA-011	Agonist	Concentric Analgesics Los Altos
	JNJ-39439335	Antagonist	Johnson & Johnson
	MR-1817	Antagonist	Mochida Pharmaceutical
	PF-3864086	Antagonist	Pfizer
	PHE-377	Antagonist	Pharmeste
	SAR-115740	Antagonist	Sanofi Aventis
*TRPV3*	SAR-292833	Modulator	Sanofi Aventis

**Table 2:** Genetic variants that, following feature selection, were included in the genotype–phenotype association, and their potential biological consequences as queried from several publicly available databases (NCBI gene index database at http://www.ncbi.nlm.nih.gov/gene, GeneCards at http://www.genecards.org, Short Genetic Variations database (dbSNP) at https://www.ncbi.nlm.nih.gov/snp, and the “1000 Genomes Browser” at https://www.ncbi.nlm.nih.gov/variation/tools/1000genomes; all accessed in January 2020). The putative functional consequences according to [34] are amino acid or protein changes for missense and deletion/insertion variants (indicated in DNA Change by *) and reduced transcriptional efficiency for untranslated region (UTR) and synonymous exonic variants. At the right of the table, the Eigen scores [35] of each variant queried from are shown, followed by the allelic frequencies at which each variant was present in the member of the two phenotypic clusters.

Gene	Rank	Variant	DNA Change^#^	Molecular Consequence	DBSNP ID	Allelic Frequency Cluster #1 [%]	Allelic Frequency Cluster #2 [%]
*ASIC1*	27	X12.50467644.SNV	G > T	INTRONIC	rs706792	32	42
*ASIC2*	3	X17.31618732.SNV	A > G	REGULATORY	rs9893935	39	66
*ASIC2*	6	X17.31619500.SNV	T>C	REGULATORY	rs9906918	48	42
*ASIC2*	17	X17.31619744.Ins	* > C	INSERTION	rs981465862	21	38
*ASIC2*	26	X17.31340390.SNV	T > C	3PRIME_UTR	rs28936	39	56
*ASIC4*	37	X2.220402680.SNV	T > C	REGULATORY	rs11695248	45	40
*TRPA1*	21	X8.72966124.SNV	A > G	DOWNSTREAM INTRONIC	rs3735944	50	28
*TRPA1*	32	X8.72966002.SNV	G > A	SYNONYMOUS	rs3735943	30	50
*TRPC1*	38	X3.142526594.SNV	T > C	DOWNSTREAM	rs4627	20	30
*TRPM3*	11	X9.73150873.SNV	T > G	NON-SYNONYMOUS	rs17535963	24	34
*TRPM3*	28	X9.73461337.SNV	T > A	SYNONYMOUS	rs7862440	35	16
*TRPM3*	29	X9.73461558.SNV	G > C	INTRONIC	rs10868907	48	62
*TRPM3*	33	X9.73457832.SNV	G > A	INTRONIC	rs1337031	39	56
*TRPM3*	34	X9.73150918.SNV	C > T	NON-SYNONYMOUS	rs41287373	43	60
*TRPM3*	35	X9.73457861.SNV	A > G	INTRONIC	rs1337030	24	36
*TRPM5*	8	X11.2435956.SNV	C > T	NON-SYNONYMOUS	rs4929982	30	48
*TRPM5*	16	X11.2435931.SNV	C > T	INTRONIC	rs4929980	20	4
*TRPM5*	22	X11.2435809.SNV	T > C	INTRONIC	rs4930102	37	44
*TRPM5*	25	X11.2435946.SNV	A > C	SPLICE_SITE	rs4929981	45	54
*TRPV1*	2	X17.3469853.SNV	A > C	3PRIME UTR	rs4790522	15	36
*TRPV1*	9	X17.3480447.SNV	T > C	NON-SYNONYMOUS	rs8065080	46	62
*TRPV2*	15	X17.16318932.SNV	T > C	REGULATORY	rs3813769	30	42
*TRPV2*	24	X17.16325968.SNV	A > G	SYNONYMOUS	rs8121	31	50
*TRPV2*	30	X17. 16323609.Del	TAGT > *	DELETION	rs5819569	43	30
*TRPV2*	36	X17.16323589.SNV	C > A	REGULATORY’	rs4273076	49	32
*TRPV3*	1	X17.3433672.SNV	C > A	REGULATORY’	rs376793	32	40
*TRPV3*	5	X17.3427442.SNV	A > G	INTRONIC	rs62069862	36	36
*TRPV3*	7	X17.3416555.SNV	C > A	INTRONIC	rs7208811	48	58
*TRPV3*	10	X17.3448331.SNV	A > T	INTRONIC	rs12945853	49	58
*TRPV3*	12	X17.3447914.SNV	C > T	SYNONYMOUS	rs1039519	46	34
*TRPV3*	18	X17.3436080.SNV	C > T	SYNONYMOUS	rs395357	43	30
*TRPV3*	19	X17.3430271.SNV	A > C	INTRONIC	rs11657715	46	62
*TRPV3*	20	X17.3415646.SNV	A > G	DOWNSTREAM INTRONIC	rs9303177	49	58
*TRPV3*	23	X17.3416309.SNV	A > G	DOWNSTREAM INTRONIC	rs9303177	32	16
*TRPV3*	31	X17.3414160.SNV	T > C	DOWNSTREAM INTRONIC	rs2271158	49	58
*TRPV4*	4	X12.110246383.SNV	T > C	INTRONIC	rs3742031	48	36
*TRPV4*	13	X12.110246369.SNV	A > G	INTRONIC	rs3742032	-	-
*TRPV4*	14	X12.110240838.SNV	T > A	NON-SYNONYMOUS	rs3825394	15	30

#: * = “none”.

**Table 3:** PubMed search results about previous evidence of a functional role of selected genetic variants.

Gene Symbol	Gene Name	PubMed Hits	Clinical Setting
*TRPA1*	Transient receptor potential channel A1	14	Erythromelalgia, miscellaneous pain, neuropathic pain, post-surgery pain, thermal pain sensitivity.
*TRPV1*	Transient receptor potential channel V1	45	Erythromelalgia, migraine, miscellaneous pain, neuropathic pain, osteoarthritis.
*TRPV2*	Transient receptor potential channel V2	1	Fibromyalgia.
*TRPV3*	Transient receptor potential channel V3	5	Fibromyalgia, migraine, miscellaneous, thermal pain sensitivity.
*TRPV4*	Transient receptor potential channel V4	2	Miscellaneous, thermal pain sensitivity.
